# Flickers of awareness: Cerebellar stimulation in disorders of consciousness

**DOI:** 10.1016/j.neurot.2025.e00636

**Published:** 2025-07-07

**Authors:** David Fischer

**Affiliations:** Department of Neurology, University of Pennsylvania, Philadelphia, PA, USA

**Keywords:** Disorders of consciousness, Transcranial magnetic stimulation, Neuromodulation

There is a growing interest in disorders of consciousness (DoC) following severe acute brain injury, and for good reason. These disorders can be devastating for the patients who suffer from them and for their families. The bulk of the literature in this field, however, is descriptive. Studies have evaluated metrics of neural dysfunction, investigated novel methods for evaluating levels of consciousness, and developed techniques to predict prognosis. There is relatively less progress in therapeutics [1], though it is not for a lack of trying. Pharmacologic stimulants have demonstrated modest promise in some specific settings, with amantadine conferring transient improvements in arousal in subacute traumatic brain injury [2]. However, such stimulants have demonstrated less efficacy more generally in DoC [3]. Deep brain stimulation has also shown preliminary promise [4], but it is an invasive approach with inconsistent outcomes. Some studies have suggested that non-invasive brain stimulation, with technologies such as transcranial magnetic stimulation (TMS) and transcranial direct current stimulation (tDCS), may also promote consciousness recovery. Early studies suggested that tDCS to the frontal lobes transiently improved consciousness [5], but these effects were not sustained in a larger cohort [6]. TMS studies have similarly yielded conflicting results, with some suggesting transient improvements in consciousness with repetitive stimulation [7]. Throughout studies of brain stimulation in DoC, there has been uncertainty about which neuroanatomical targets to stimulate, and it has been postulated that variable treatment responses may be related to improper targeting.

In this issue of Neurotherapeutics, Wen Jiang and colleagues investigated the application of TMS to the cerebellum in patients with DoC [8]. They justified this unconventional target by arguing that lobule VII of the cerebellum demonstrates connectivity with the frontoparietal network, which has been associated with consciousness in prior studies. They used a randomized, sham-controlled, double-blinded, cross-over trial design – a thoughtful design that reflects the challenges of recruiting and retaining this patient population. Their primary outcome measure was the Coma Recovery Scale - Revised (CRS-R), a gold standard behavioral assessment of consciousness, after five consecutive sessions of TMS. Their secondary outcomes were CRS-R scores after the first session, changes in EEG patterns, and longer-term recoveries. The trial did not achieve its primary endpoint, with no difference in consciousness after a full treatment session. However, there were transient improvements in CRS-R and EEG complexity after individual TMS sessions.

Their findings echo those from many prior studies: the possibility of transient improvements without enduring effects. Why is this such a common result? States of consciousness in DoC are known to fluctuate substantially: arousal can vary between days, hours, or minutes. The physiology of such fluctuations is uncertain, but it is plausible that the physiology underlying longer-term consciousness recovery (perhaps the product of structural plasticity) is distinct from the physiology underlying shorter-term fluctuations (perhaps the product of arousal or changes in synaptic transmission). It may similarly be the case that interventions such as TMS, amantadine, or other transient therapeutics modulate the latter and not the former. There may be clinical benefits to transient improvements – it may provide more opportunities for communication with the patient, or a glimpse into what levels of consciousness they are capable of. But certainly enduring improvements that alter the long-term trajectory of recovery is the ultimate objective.

The study's primary strength is in its aim to identify a therapeutic target in DoC, which has to date remained elusive. The study's trial design was rigorous, and their broad inclusion criteria optimized generalizability. There are also limitations. It is difficult to devise an appropriate sham condition for TMS, a drawback of therapeutic TMS research more generally. The researchers rotated the coil 90°, a common sham strategy, but doing so eliminates the somatosensory stimulation which could be transiently arousing in patients with DoC, raising the possibility of confounding. The long-term outcome measures they studied were also unconventional, as their phone-based consciousness assessments had not been previously validated.

As for the therapeutic studies before it, these findings warrant further investigation. Identifying the mechanisms by which this intervention causes improvements, albeit transiently, may lend insights into how to produce more sustained improvements. If, for example, the potentially therapeutic effect of cerebellar stimulation is via indirect stimulation of the frontoparietal network, then perhaps targeting this network more directly would yield greater benefit. Ultimately, this study represents a step in the right direction for the field – to try, even if imperfectly, to not merely describe and prognosticate in DoC, but to intervene to improve outcomes.

## Declaration of competing interest

The authors declare the following financial interests/personal relationships which may be considered as potential competing interests: David Fischer reports financial support was provided by National Institute of Neurological Disorders and Stroke. David Fischer reports financial support was provided by American Academy of Neurology. David Fischer reports financial support was provided by Neurocritical Care Society. If there are other authors, they declare that they have no known competing financial interests or personal relationships that could have appeared to influence the work reported in this paper.
